# *Drosophila CG2469* Encodes a Homolog of Human CTR9 and Is Essential for Development

**DOI:** 10.1534/g3.116.035196

**Published:** 2016-09-27

**Authors:** Dhananjay Chaturvedi, Mayu Inaba, Shane Scoggin, Michael Buszczak

**Affiliations:** Department of Molecular Biology, University of Texas Southwestern Medical Center, Dallas, Texas 75390

**Keywords:** germline, PAF1 complex, chromatin, RNAi, Ctr9

## Abstract

Conserved from yeast to humans, the Paf1 complex participates in a number of diverse processes including transcriptional initiation and polyadenylation. This complex typically includes five proteins: Paf1, Rtf1, Cdc73, Leo1, and Ctr9. Previous efforts identified clear *Drosophila* homologs of Paf1, Rtf1, and Cdc73 based on sequence similarity. Further work showed that these proteins help to regulate gene expression and are required for viability. To date, a *Drosophila* homolog of Ctr9 has remained uncharacterized. Here, we show that the gene *CG2469* encodes a functional *Drosophila* Ctr9 homolog. Both human and *Drosophila* Ctr9 localize to the nuclei of *Drosophila* cells and appear enriched in histone locus bodies. RNAi knockdown of *Drosophila Ctr9* results in a germline stem cell loss phenotype marked by defects in the morphology of germ cell nuclei. A molecular null mutation of *Drosophila Ctr9* results in lethality and a human cDNA CTR9 transgene rescues this phenotype. Clonal analysis in the ovary using this null allele reveals that loss of *Drosophila Ctr9* results in a reduction of global levels of histone H3 trimethylation of lysine 4 (H3K4me3), but does not compromise the maintenance of stem cells in ovaries. Given the differences between the null mutant and RNAi knockdown phenotypes, the germ cell defects caused by RNAi likely result from the combined loss of *Drosophila Ctr9* and other unidentified genes. These data provide further evidence that the function of this Paf1 complex component is conserved across species.

The RNA Polymerase II-associated complex (Paf1 complex) was identified in yeast based on its physical association with RNA Polymerase II (Pol II) (Shi *et al.* 1996). Subsequent work showed that Paf1 associates with four other proteins: Rtf1, Leo1, Cdc73, and Ctr9 (RNA polymerase-associated protein CTR9 homolog) (Krogan *et al.* 2002). The human Paf1 complex contains a sixth protein, Ski8 (Zhu *et al.* 2005). The Paf1 complex participates in multiple aspects of transcription across species. For example, Paf1 complex members promote transcriptional elongation of the Pol II complex by recruiting Rad6 and Bre1 (Krogan *et al.* 2003), which target histone H2B for mono-ubiquitylation. In turn, the presence of ubiquitylated H2B promotes the trimethylation of histone H3 on lysine 4 (H3K4me3) and Pol II elongation (Kim *et al.* 2009). In addition to its role in regulating transcriptional elongation, the Paf1 complex has also been implicated in telomeric silencing (Ng *et al.* 2003), 3′ end formation of mRNA (Krogan *et al.* 2003; Li *et al.* 2003; Schaft *et al.* 2003), and 3′ end formation of snoRNAs (Tomson *et al.* 2013). Other studies suggest that the Paf1 complex may also participate in transcriptional repression in certain contexts (Crisucci and Arndt 2011, 2012; Yoo *et al.* 2014).

To date, several components of the *Drosophila* Paf1 complex have been identified based on sequence homology (Adelman *et al.* 2006; Tenney *et al.* 2006). *Drosophila* Paf1, Rtf1, and Cdc73 colocalize with each other and with active Pol II (Adelman *et al.* 2006). Furthermore, these proteins are recruited to heat shock response genes upon heat stress. Knockdown of Paf1 results in diminished recruitment of Spt6 and FACT, suggesting that the *Drosophila* Paf1 complex may regulate active transcription by helping to alter chromatin structure (Adelman *et al.* 2006). Subsequent work has shown that components of the *Drosophila* Paf1 complex interact with a number of signaling pathways. For example, Cdc73 (also known as hyrax and parafibromin) promotes Wnt signaling through physical association with β-catenin (Mosimann *et al.* 2006). Disruption of Cdc73 also attenuates hedgehog signaling (Mosimann *et al.* 2009). In addition, Rtf1 and the Paf1 complex-associated ubiquitin ligase Bre1 promote Notch signaling in a number of different contexts (Bray *et al.* 2005; Tenney *et al.* 2006; Akanuma *et al.* 2007). More recent work suggests that *Drosophila* Bre1 regulates both germline stem cell (GSC) maintenance and germline cyst development in the ovary (Xuan *et al.* 2013). However, the function of the Paf1 complex during oogenesis remains uncharacterized.

In contrast to Paf1, Rtf1, and Cdc73, no clear *Drosophila* homologs of Ctr9 and Leo1 have been described in the literature. Here, we identify the previously uncharacterized gene *CG2469* as the *Drosophila Ctr9* homolog based on sequence homology. A molecular null mutation of *Drosophila Ctr9* generated through homologous recombination exhibits embryonic and early larval lethality. The lethality caused by loss of *Drosophila Ctr9* can be rescued by expression of both *Drosophila* and human *Ctr9* cDNA transgenes, providing evidence that the function of this Paf1 complex component has been conserved across species.

## Materials and Methods

### Fly stocks

The following *Drosophila* stocks were used in this study: daughterless-Gal4 (da-Gal4), nanos-Gal4::VP16, and hs-FLP; histoneGFP FRT2A/TM3. y^1^ w*/Dp(2;Y)G, P{hs-hid}Y; P{70FLP}11 P{70I-SceI}2B noc^Sco^/CyO, P{hs-hid}4 (BL#:25680), Df(3L)BSC250/TM6C (BL#23150), and Ctr9^RNAi-3^ (BL#:33736) were obtained from the Bloomington Stock Center. The human rescuing transgenic line was made by PCR amplifying the *CTR9* ORF from the cDNA construct NM_014633 (Origene) (see Supplemental Material, Table S1 for primers). The resulting PCR product was cloned into a pPHW vector, which contains a phiC31 AttB site, using the Gateway LR reaction following the manufacturer’s instructions (Invitrogen). The resulting vector was sequence verified and transformed into the M{3xP3-RFP.attP}ZH-2A landing site of *Drosophila* embryos using phiC31-mediated recombination (Rainbow Transgenics).

### Antibodies

The following antibodies were used in this study: Rabbit anti Lsm11 (1:2000, gift from Joseph Gall), Rabbit polyclonal anti-GFP (1:1000 Invitrogen), Mouse anti-HA (1:100 for IHC, Covance), Rat anti-HA (1:1000 for WB, Roche), Mouse anti-Flag (1:2000 for WB, Invitrogen), Mouse anti-Hts (1:20, 1B1 DSHB Iowa), and Rabbit anti-H3K4me3 (1:1000 Upstate). Cy3, Cy5, FITC (Jackson Laboratories), or Alexa 488 (Molecular Probes) fluorescence-conjugated secondary antibodies were used at a 1:200 dilution.

Sequence corresponding to the first 200 amino acids of CG2469 was cloned into the pDEST17 Gateway^TM^ vector (Invitrogen) to produce a 6 × His-tagged protein. The protein was expressed in BL21-AITM *Escherichia coli* (Invitrogen) and purified with Ni-NTA agarose (Invitrogen) under denaturing conditions. Polyclonal antisera were generated in two guinea pigs, TX1010 and TX1011 (Covance). All the experiments described here were performed with antiserum from TX1011.

### Immunohistochemistry

Briefly, dissected adult ovaries were fixed with 4% formaldehyde in 1 × PBS for 10 min. After fixation, the ovaries were washed three times with PBSTA (1 × PBS, 0.4% Triton X-100, and 0.5% BSA) for 10 min. The tissue was then incubated with the appropriate antibodies overnight at 4°. After 8–10 min washes at room temperature (RT), the samples were incubated with secondary antibodies for 4 hr at RT. The samples were then washed three times in PBSTA for 10 min each and mounted in Vectashield with DAPI. Imaging was performed with Zeiss Lsm510 and Zeiss Lsm710 microscopes.

### Generation of the Ctr9 knockout

The *Ctr9* knockout allele (*Ctr9^KO^*) was generated by the method described previously (Chan *et al.* 2011). First, homology arms ∼500 bp in length were amplified off of the P[acman] clone 38M10 (CHORI). CG2469Ctr-9LA-Fv2 and CG2469Ctr-9LA-R primers (Table S1) were used to amplify the left homology arm, 7.5 kb upstream of the CG2469 CDS, and CG2469Ctr-9RA-F and CG2469Ctr-9RA-Rv2 primers were used to amplify the right arm, 4.3 kb downstream of the CG2469 CDS. These PCR products were stitched together through a PCR-SOE reaction using the CG2469Ctr-9LA-Fv2 and CG2469Ctr-9RA-Rv2 primers. The resulting 1 kb PCR product was cloned into the P[acman] KO vector using a Gateway BP reaction following the manufacturer’s instructions (Invitrogen BP clonase II 11789-020). This vector was then linearized using BAMHI-HF (NEB R3136S) at 37° for 6 hr, subjected to gel electrophoresis, and gel extracted using a Zymoclean Gel DNA recovery kit (Zymo Research D4008). This DNA was then electroporated into recombineering-competent DY380 cells carrying the 38M10 clone. Resulting recombinant clones were subjected to PCR analysis using LA and RA check primers (Table S1) to verify the LA and RA homology arm junctions. Clones passing this test were subjected to a second round of recombineering designed to replace the *Ctr9* gene with a 3XP3/Kan cassette. This replacement cassette was generated by amplifying the previously described 3XP3/Kan cassette (Chan *et al.* 2011, 2012) using the Ctr9KO forward and Ctr9KO reverse primers (Table S1). This complete *CG2469/Ctr9* knockout vector was sequence verified and then transformed into *Drosophila* embryos using phiC31-mediated recombination in combination with the M{3xP3-RFP.attP’}ZH-68E landing site.

The KO cassette was mobilized by the induction of *hs*-Flp and *hs*-I-SCEI. New RFP-positive insertions were balanced and crossed to the *Df(3L)BSC250* deficiency, which uncovers the *CG2469* locus. One out of 37 potential knockouts exhibited lethality over the deficiency. This line was verified as a knockout allele of the *Ctr9* gene using Southern blotting (described below) and rescue experiments. This *Ctr9^KO^* allele was recombined onto an isogenized *FRT2A* chromosome for clonal analysis.

### Identification of stage of lethality

Fifty fertilized *Ctr9^KO^*/TM3 *Kr*-GFP females were placed on grape juice agarose plates with wet yeast at RT. After 72 hr, 250 larvae were scored for GFP fluorescence under a Zeiss Discovery V8 microscope.

### Mosaic analysis

*Ctr9^KO^* homozygous clones were generated by FLP/FRT-mediated mitotic recombination. Adult *hs-FLP*; *FRT2A histoneGFP/FRT2A Ctr9^KO^* females were heat-shocked at 37° for 1 hr twice a day for 3 d; *hs-FLP*; *FRT2A histoneGFP/FRT2A +* flies were used as controls. Ovaries were dissected at the indicated times after clone induction. The number of GFP-negative *Ctr9^KO^* homozygous GSC clones was determined.

### Southern blotting

Briefly, genomic DNA was extracted from *y w* control, donor, and *Ctr9^KO^*/TM3 flies and digested with *Xho*I. Each digest was split into two parts and run on a 4% agarose gel. The gel was incubated in Denaturing solution (1.5 M NaCl and 0.5 M NaOH in water) for 45 min, followed by Depurinating solution (0.2 M HCl) for 15 min, rinsed several times in distilled water, then incubated in Neutralizing solution (1 M Tris, pH 7.4, 1.5 M NaCl, and ∼70 ml 37% HCl) for 30 min. The DNA was transferred onto nitrocellulose and cross-linked using standard protocols. The membrane was incubated in preheated hybridization buffer for 30 min at 42°, after which DIG-labeled probe was added and the membrane was incubated overnight at 42°. The membrane was washed twice in 2 × SSC; 0.1% SDS at RT, then washed twice in 0.5 × SSC; 0.1% SDS at 68°, rinsed in maleic acid buffer for 5 min, and blocked in 1% blocking buffer (Roche, #11 096 176 001) in maleic acid for 1–3 hr at RT. Anti-DIG antibody (Roche, #11 093 274 910) was diluted 1:10,000 in fresh blocking buffer and incubated with the membrane for 30 min at RT with gentle shaking. The membrane was then washed for 2 × 15 min in wash buffer (30 ml Maleic acid buffer and 90 μl Tween 20), rinsed in detection buffer (100 ml 1 M Tris pH 9.5 and 20 ml 5 M NaCl) for 5 min. CDP-Star solution (Applied Biosystems T2146) was used for detection.

Arm specific probes were generated using the following primer sets. Left arm: forward, 5′-GGCCATTGACGAATGGAGTT-3′; reverse, 5′-AGGACCACAAGGCACTGGAA-3′. Right arm: forward, 5′-TGGTACCCATCCGTCGGTAG-3′; reverse, 5′-TGACCATGTGACGTGCTTCC-3′. The resulting PCR products were labeled using a DIG labeling mix (Roche, #11 835 289910) in combination with Klenow (NEB, #M0212L).

### Generation of RNAi lines

RNAi lines *Ctr9^RNAi-1^* and *Ctr9^RNAi-2^* targeting *CG2469* were generated according to TRiP protocols (Ni *et al.* 2011) using the si1CG2469 and si2CG2469 oligos listed in Table S1. The two resulting plasmids were transformed into *Drosophila* using phiC31-mediated integration at M{3xP3-RFP.attP’}ZH-68E.

### Statistical analysis and graphing

Statistical analysis and graphing were performed using Microsoft Excel software. Data are shown as means and SD. The *P* value (two-tailed Student’s *t*-test) is provided for comparison with the control.

### Data availability

The authors state that all data necessary for confirming the conclusions presented in the article are represented fully within the article.

## Results

### CG2469 encodes a putative homolog of human CTR9

**Figure 1:**
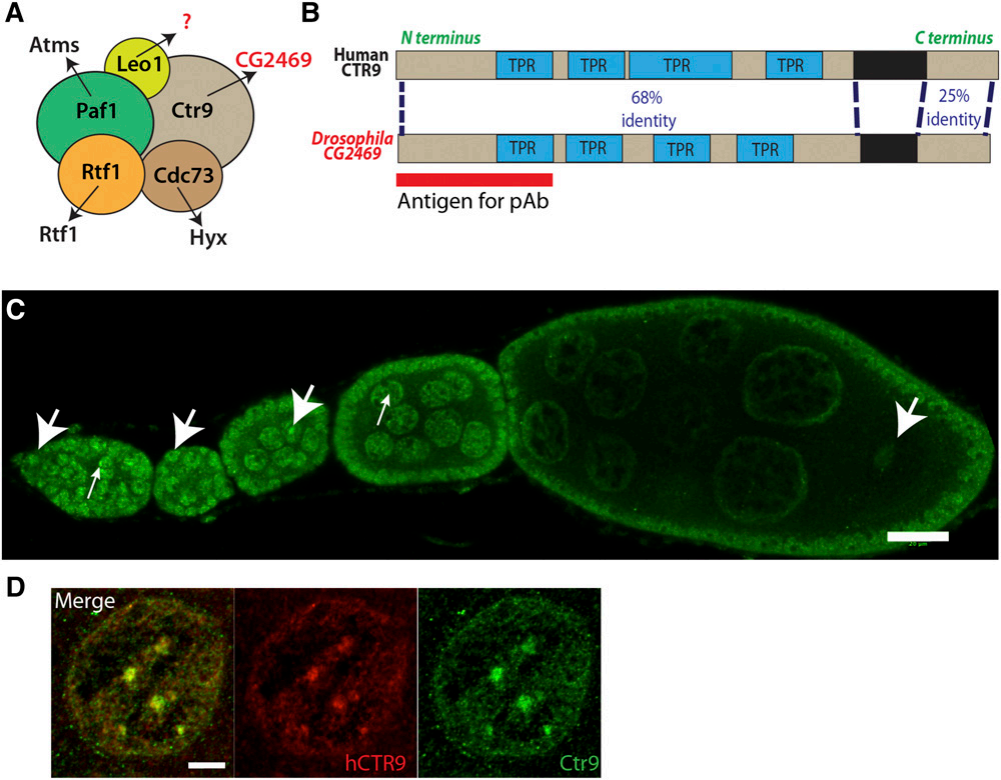
CG2469 is the *Drosophila* homolog of human CTR9. (A) The known members of the Paf1 complex in yeast through humans include Paf1, Cdc73, Rtf1, Ctr9, and Leo1. (B) CG2469 and human CTR9 have four TPR domains, sharing 68% identity through this region. The red bar denotes the first 200 amino acids of CG2469 employed to raise polyclonal sera. (C) CG2469 is expressed in all cell types (arrowheads) in ovaries. Nuclear CG2469 is enriched in punctae on chromatin in all cell types including nurse cells (small arrows). (D) HA-tagged human CTR9 driven by *nanos*-Gal4 colocalizes with endogenous CG2469 in germline stem cells, including within bright punctae on chromatin. Scale bars, (C) 20 µm; (D) 5 µm. TPR, tetratricopeptide.

Studies using yeast and human cell lines revealed a key role for the Paf1 complex component Ctr9 in chromatin modification and polyadenylation (reviewed in Tomson and Arndt 2013). However, up until now, a *Drosophila* homolog of Ctr9 has not been described in the literature. Given the conserved functions of the Paf1 complex across species, we reasoned that the *Drosophila* genome likely encodes a Ctr9 homolog. Sequence homology searches revealed that the predicted protein product of *CG2469* shares an overall amino acid identity of 53% with human CTR9 (Figure 1, A and B). Like its human counterpart, CG2469 possesses multiple tetratricopeptide repeats that likely provide a binding surface for interaction with other proteins in the Paf1 complex (Figure 1B). Given this sequence homology and genetic analyses described below, we will refer to *CG2469* as *Ctr9* hereafter.

**Figure 2:**
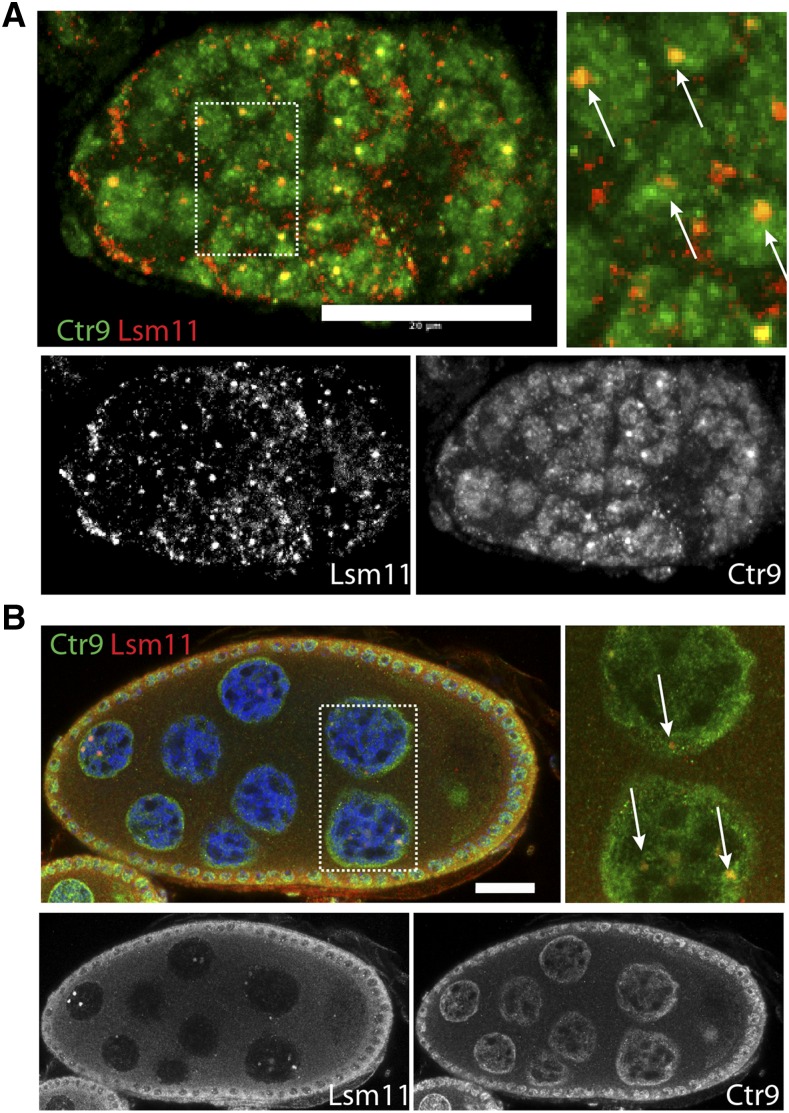
*Drosophila* Ctr9 and the human *CTR9* transgene are enriched at Histone Locus Bodies. (A and B) Lsm11 (red), a Histone Locus Body marker, colocalizes with Ctr9 (green) enrichment sites within nuclei of germ cells (arrows). Yellow dots arise from overlap of red and green punctae. Magnified views of the boxed regions are shown on the right panels. Scale bars, 20 µm.

Based on previous results that showed the importance of Bre1 during the early stages of *Drosophila* oogenesis (Xuan *et al.* 2013), we were particularly interested in analyzing the function of Ctr9 in the ovary. As a first step toward determining the expression pattern and subcellular localization of *Drosophila* Ctr9, we generated a polyclonal antibody against the N-terminus of the protein. Staining wild-type ovaries revealed that Ctr9 exhibited ubiquitous expression throughout the ovary and largely localized to the nucleus, as expected (Figure 1C). This antibody appeared specific in that it did not label Ctr9 mutant cells (see below). While we observed Ctr9 staining throughout the nucleus, the protein sometimes appeared enriched in a small nuclear body (Figure 1, C and D and Figure 2, A and B). To determine whether human CTR9 exhibited a similar localization pattern, we generated a HA-tagged human *CTR9* transgene under control of the UAS promoter. We then expressed this transgene specifically in the germline using a *nanos (nos)-Gal4* driver. Colabeling these ovaries for the human CTR9 transgene and endogenous *Drosophila* Ctr9 revealed that the two proteins displayed virtually identical localization patterns, further suggesting that these proteins share potential functional homology (Figure 1D). Importantly, the antibody directed against *Drosophila* Ctr9 did not cross-react with transgenic human CTR9 (Figure S1).

To more fully characterize the subnuclear localization of Ctr9, we colabeled wild-type ovaries for *Drosophila* Ctr9 and a variety of other markers that labeled various nuclear subdomains, including the nucleolus, cajal bodies, and histone locus bodies. Ctr9 did not display convincing colocalization with most of these markers. By contrast, we observed discrete colocalization between Ctr9 and Lsm11 within some histone locus bodies in the germline (Figure 2, A and B). This colocalization was most easily seen in germaria, but could also be occasionally observed in maturing egg chambers. Ctr9 has not previously been implicated in the transcription or processing of histone RNA. Further analysis will be required to determine the significance of Ctr9 enrichment in the histone locus body.

### The Ctr9 RNAi knockdown results in nuclear defects within germ cells

**Figure 3:**
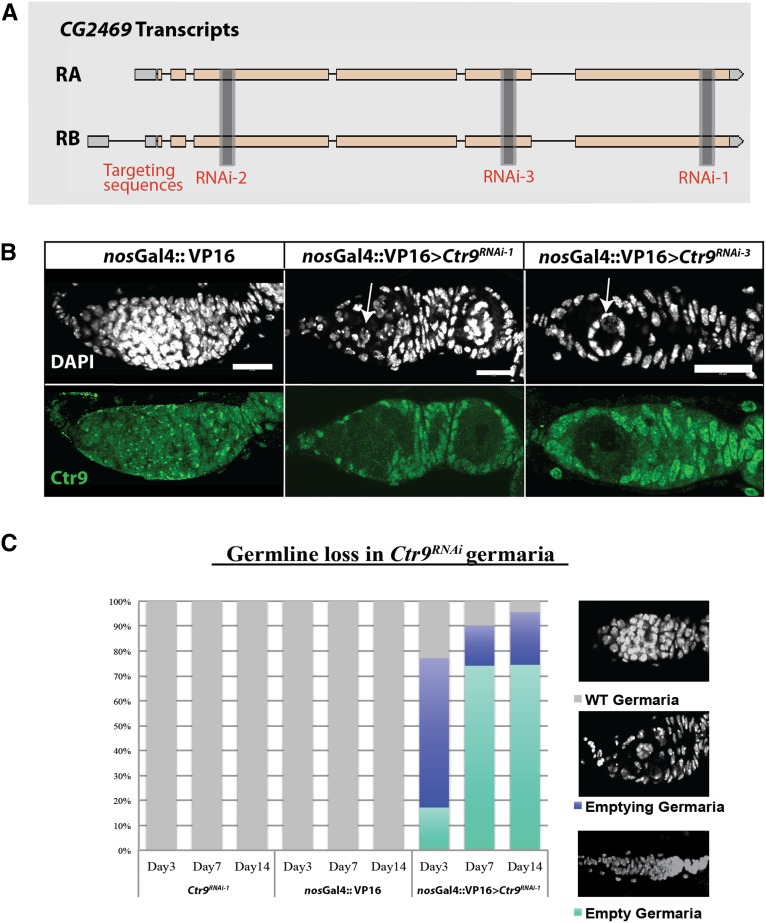
RNAi targeting *Ctr9* in the germline. (A) A schematic of two isoforms of *CG2469* (RA and RB) and three independent RNAi sequences targeting different exons of the gene. (B) DAPI staining in *nos*-Gal4 control germaria show normal germline nuclear size and WT Ctr9 expression (green); *Ctr9^RNAi-1^* (column 2) and *Ctr9^RNAi-3^* (column 3) driven by *nos*-Gal4 have polyploid cells in the germarium (DAPI). Examples shown here are representative images from day 7 and day 1 for both lines, respectively. Both lines show loss of Ctr9 (green channel in column 2,3). (C) Quantification of germline loss in *Ctr9^RNAi-1^* animals over time. Control animals of parent stocks *Ctr9^RNAi-1^* and *nos*-Gal4 show no polyploidy or germline loss over time. Germaria from *nos-*Gal4 *> Ctr9^RNAi-1^* females lose germ cells over time. By day 21, all germaria are devoid of germ cells. Scale bars, 20 μm. DAPI, 4’,6-diamidino-2-phenylindole; RNAi, RNA interference; WT, wild type.

To foster our efforts to characterize the function of Ctr9, we created two UAS-RNAi lines (*Ctr9^RNAi-1^* and *Ctr9^RNAi-2^*) that could be used in combination with cell-specific Gal4 drivers. These constructs were designed to target specific and unique sequences within the annotated *Ctr9* transcripts (Figure 3A) and were made with the recently developed Valium 20 vector (Ni *et al.* 2011), which allows for germline expression of RNAi transgenes. We also analyzed a third *Ctr9* RNAi transgenic line, publicly available from the Bloomington stock center (*Ctr9^RNAi-3^*). Ubiquitous expression of all three RNAi lines driven by *daughterless-Gal4* caused lethality, consistent with previous loss-of-function studies of other members of the Paf1 complex. As expected, driving the expression of all three of these RNAi constructs specifically in the germline using a *nos*-Gal4 driver resulted in the reduction of Ctr9 expression (Figure 3B). Strikingly, the expression of these constructs also resulted in pronounced morphological defects within germ cell nuclei (Figure 3B). These nuclei appeared much larger than those of control cells, suggesting that they may be polyploid. The appearance of multiple Lsm11-positive loci within these cells further suggested that RNAi knockdown of *Ctr9* results in aberrant polyploidization of germ cells (data not shown). This phenotype was consistent across all three RNAi lines (Figure 3B and Figure S2, A and B). In animals where *Ctr9* was knocked down using *Ctr9^RNAi-1^*, cells with aberrant nuclear morphology were observed in the germaria beginning 3 d after eclosion. This phenotype worsened over time and eventually led to a complete germ cell loss by 21 d post eclosion (Figure 3C). The rate of germ cell loss varied between the three lines. While most germaria from *Ctr9^RNAi-1^* and *Ctr9^RNAi-3^* exhibited defects after eclosion, *Ctr9^RNAi-2^* began to display similar phenotypes 2 wk after the flies had eclosed. To test the specificity of the RNAi phenotype, we coexpressed the human *CTR9* transgene in combination with the *Ctr9^RNAi^* constructs. The nucleotide sequence of the *Drosophila* and human *Ctr9* genes exhibit significant divergence so that the *Drosophila Ctr9* RNAi constructs should not target the human cDNA transgene. Indeed, the expression of the human CTR9 HA-tagged transgene rescued the nuclear phenotypes associated with RNAi knockdown of *Drosophila Ctr9* (Figure S3).

### Generation of a Drosophila Ctr9 null mutation

**Figure 4:**
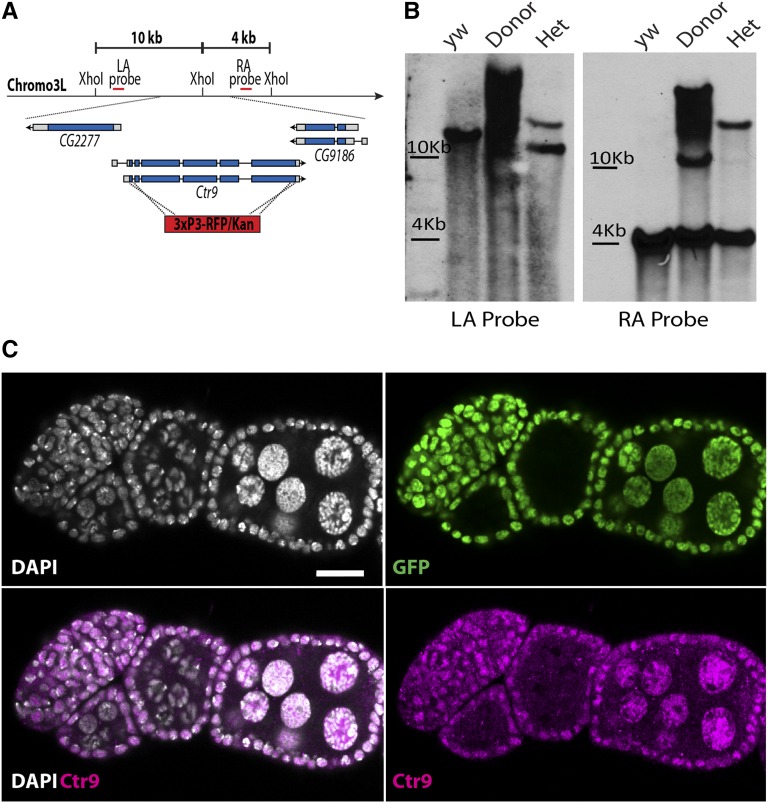
*Ctr9* knockout clones show a loss of Ctr9 protein. (A) A schematic of the *Ctr9* locus showing the strategy used to generate the *Ctr9^KO^* allele. 7.5 and 4.3 kb homology arms on either side of the RFP/Kan cassette were employed to specifically target *Ctr9*. A *Xho*I site exists in the genomic sequence but not the RFP/Kan cassette. (B) Both homology arms were probed for on a Southern blot from *Xho*I restriction digested genomic DNA from *y w* controls, donor controls, and heterozygous *Ctr9^KO^* flies. Control *Xho*I digested genomic DNA runs at 10 kb (left arm) and 4 kb (right arm). Chromosomal DNA bearing the KO cassette, in both cases, runs at 15.4 kb. (C) Wild-type (*histone*GFP-positive) egg chambers show Ctr9 (magenta) staining in nurse cell nuclei. Clonal egg chambers (*histone*GFP-negative) do not. Scale bars, 20 μm. DAPI, 4’,6-diamidino-2-phenylindole; GFP, green fluorescent protein; Kan, Kanamycin; KO, knockout; LA, left arm; RA, right arm. RFP, red fluorescent protein.

To more fully characterize the genetic function of this gene, we elected to generate a molecularly defined *Ctr9* null allele using Bac-based recombineering (Chan *et al.* 2011; Carreira-Rosario *et al.* 2013). We generated a knockout vector that replaced the 3.6 kb coding sequence shared by both annotated isoforms of *Ctr9* with a 3XP3/KAN selectable cassette (Figure 4A). This vector, which included homology arms approximately 7 kb upstream and 4 kb downstream of *Ctr9* was inserted into a landing site on the second chromosome. The cassette was then mobilized from the landing site by inducing the expression of FLPase and I-Sce1. New insertions of the knockout cassette on the third chromosome were selected. Out of 37 lines screened, only one failed to complement the *Df(3L)BSC250* deficiency, which uncovers the *Ctr9* locus. This putative *Ctr9^KO^* allele was itself homozygous lethal, with only 1% of larvae surviving to second instar. We used Southern blot analysis to verify the appropriate integration of the KO cassette into the endogenous *Ctr9* locus (Figure 4B). In addition, antibody staining revealed that endogenous Ctr9 protein was clearly absent in homozygous mutant clones of the *Ctr9^KO^* allele (Figure 4C). The lethal phenotype associated with *Ctr9^KO^/Df(3L)BSC250* was rescued by a *Drosophila Ctr9* cDNA transgene under control of the *daughterless* promoter. Moreover, a human HA-tagged *Ctr9* transgene rescued the lethal phenotype caused by loss of *Ctr9*, providing further evidence that many aspects of Ctr9 function have been conserved from flies to humans (Figure S1). However, the *Ctr9^KO^/Df(3L)BSC250* adult mutant females rescued by the human transgene remained sterile, despite their ability to lay eggs. These observations indicate that some aspects of Ctr9 function within the ovary have partially diverged between *Drosophila* and humans. Further experiments will be required to fully characterize the cause of this sterility.

### The loss of Ctr9 does not affect germline and follicle stem cell (FSC) maintenance in Drosophila ovaries

**Figure 5:**
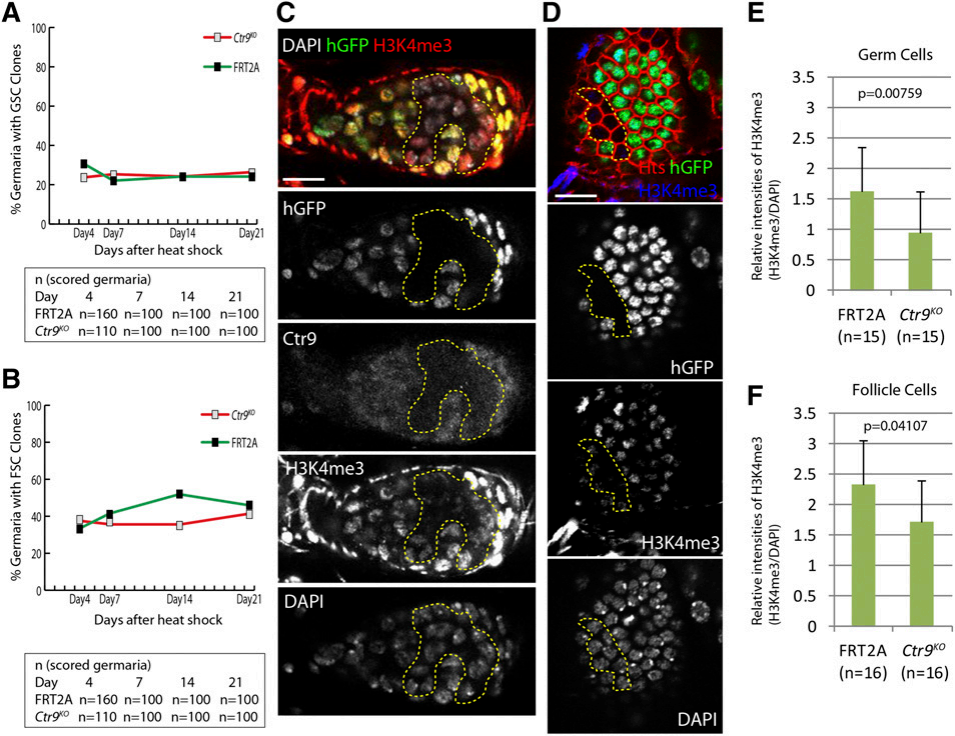
*Ctr9^KO^* clones do not exhibit GSC or FSC loss phenotypes. The percentage of germaria bearing (A) GSC and (B) FSC *Ctr9^KO^* clones were counted over a period of 21 d. *Ctr9^KO^* clonal stem cells persisted in germaria at levels similar to FRT2A control clones. (C and D) Representative images of H3K4me3 (red) staining in *Ctr9* mutant germ cell clones (C) or follicle cell clones (D). *Ctr9* mutant clones were negatively marked by the absence of hGFP. (E and F) Quantification of H3K4me3 intensity in the *Ctr9* mutant germ cell clones (E) or follicle cell clones (F). FRT2A control clones were scored for the control. Data were normalized to DAPI intensity scored from the same cell. (C–F) Ovaries were dissected at day 14 after heat shock. Scale bars, 10 µm. DAPI, 4’,6-diamidino-2-phenylindole; FSC, follicle stem cells; GSC, germline stem cells; hGFP, *histone* green fluorescent protein.

A previous study found that loss of *Bre1*, a E3 ubiquitin ligase that often functions with the Paf1 complex to regulate transcription, results in a GSC loss phenotype, marked by a dramatic reduction in the global levels of H3K4me3 (Xuan *et al.* 2013). Similarly, we also found that loss of *Bre1* results in a GSC loss phenotype (data not shown). Previous studies linked Bre1 activity to the Paf1 complex (Wood *et al.* 2003, 2005; Kim and Roeder 2009). To test if *Ctr9* is also required for GSC maintenance in a cell autonomous manner in ovaries, we counted the percentage of germaria bearing marked GSCs over a period of 21 d after clone induction. Surprisingly, *Ctr9* mutant germline stem cells persisted at levels similar to control clones over the time course of the experiment (Figure 5A). Moreover, differentiating mutant germline clones initially appeared morphologically normal up until midoogenesis, when they exhibited an egg chamber degeneration phenotype (Figure S4).

Next, we tested whether Ctr9 carries out a critical function in somatic follicle cells. We induced both control and *Ctr9^KO^* clones in the follicle cells using FLP/FRT mediated recombination and counted the percentage of germaria that contain FSC clones 4, 7, 14, and 21d after clone induction. This analysis revealed that loss of *Ctr9* does not affect the maintenance of FSCs (Figure 5B). Furthermore, we did not note any striking phenotype within *Ctr9^KO^* follicle cell clones of developing egg chambers, suggesting that Ctr9 function was dispensable within this lineage under normal conditions.

As noted above, loss of Bre1 leads to a global reduction in H3K4me3 levels in the germline, and this observation suggests that the regulation of this chromatin modification may be critical for GSC maintenance (Xuan *et al.* 2013). Given that the Paf1 complex helps to recruit Bre1 to specific genomic sites in other systems, we next tested the extent to which loss of *Ctr9* affected H3K4me3 levels within different cell types of the ovary. *Ctr9* mutant germline clones display a modest global reduction in H3K4me3 levels compared to control cells within the same samples (Figure 5, C and E). *Ctr9* mutant follicle cell clones also exhibited a similar minor reduction of anti-H3K4me3 staining (Figure 5, D and F). Together, these results indicate that loss of *Ctr9* has a minor impact on global levels of H3K4 trimethylation.

### Timing of Ctr9 loss does not account for differences between the Ctr9^RNAi^ and Ctr9^KO^ phenotypes

Expression of *Ctr9^RNAi^* results in nuclear defects within early germ cells of the germarium, leading to a loss of germ cells over time. By contrast, *Ctr9^KO^* clones are maintained over time and do not exhibit nuclear defects within the germarium. Given that *Ctr9* mutant clones were induced in adults, differences between the RNAi and null mutant phenotypes could be due to the timing of the loss of *Ctr9* function. We took a number of different approaches to test this possibility.

**Figure 6:**
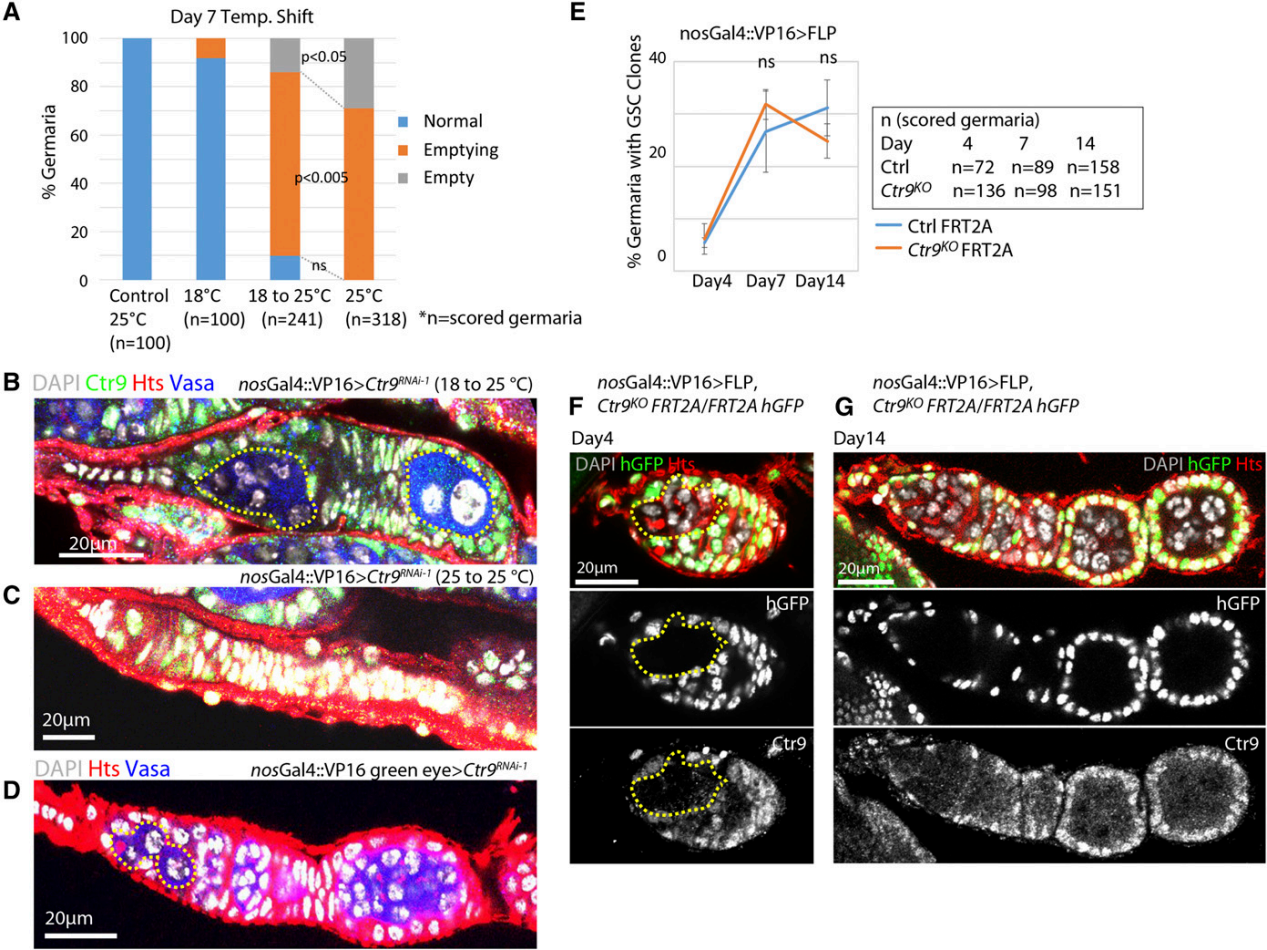
Timing does not account for differences between the *Ctr9^RNAi^* and *Ctr9^KO^* phenotypes in the germline. (A) Comparison of *Ctr9^RNAi-1^* knockdown phenotype in various temperature settings. The percentage of normal, empty, or emptying germaria were scored (*n* = number of scored germaria). Control animals show no germline loss. Only a small percentage of *nosGal4*::*VP16 > UAS-Ctr9^RNAi-1^* germaria raised and maintained at 18° show a germ cell loss phenotype. Germaria from *nosGal4*::*VP16 > UAS-Ctr9^RNAi-1^* females raised at 18° and shifted to 25° for 7 d after eclosion (18–25°) exhibit severe germ cell loss similar to the samples raised and kept at 25°. (B and C) Representative images of the germ cell loss phenotype of *nosGal4*::*VP16 > UAS-Ctr9^RNAi-1^* at each temperature setting show emptying (B) or empty (C) germaria. (D) *UAS-Ctr9^RNAi-1^* driven by an independent Gal4 driver (nosGal4::VP16 green eye) results in a germ cell loss phenotype. Enlarged germ cells are circled by yellow dotted lines in (B and D). (E) The percentage of germaria bearing *Ctr9^KO^* or control clones (genotypes as described) were counted over a period of 14 d after eclosion. *Ctr9^KO^* clonal stem cells persisted in germaria at levels similar to FRT2A control clones. (*n* = scored germaria). (F and G) Representative images of *Ctr9^KO^* clones (yellow dotted line) induced by *nosGal4*::*VP16 > UAS-FLP*. These samples do not show germ cell loss phenotypes at day 4 (F) or day 14 (G) after eclosion. Error bars indicate SD. *Ctr9^KO^* mutant clones were negatively marked by the absence of hGFP. Scale bars, 20 µm. DAPI, 4’,6-diamidino-2-phenylindole; hGFP, *histone* green fluorescent protein.

First, we varied the timing of *Ctr9^RNAi^* induction by taking advantage of the reduced efficiency of Gal4-dependent transcriptional induction at lower temperatures. We crossed the *nos-Gal4* driver to a line carrying *UAS-Ctr9^RNAi-1^*. The majority of germaria from the resulting females did not exhibit a phenotype when maintained at 18° during both development and adulthood (Figure 6A), indicating that *Ctr9* is not effectively knocked down under these conditions. In parallel, we maintained a second population of *nos-Gal4*>*UAS-Ctr9^RNAi^* females at 18° during development but shifted the adults to 25° immediately after eclosion for 7 d (Figure 6A). Germ cells from the temperature-shifted females exhibited pronounced nuclear morphological defects resulting in a germ cell loss phenotype, similar to females raised during development and maintained as adults at 25° (Figure 6, A–C). These results indicated that expression of *Ctr9^RNAi^* specifically within adults results in the observed phenotypes.

Next, we wanted to rule out the possibility that the specific Gal4 driver we had been using in our experiments somehow accounted for the difference between the RNAi and knockout phenotypes. We tested a second, independently derived *nos-Gal4* driver marked by 3 × P3-GFP (nos-Gal4::VP16 green eye). Driving *Ctr9^RNAi^* using this Gal4 line also resulted in the appearance of polyploid germ cells within germaria and a germ cell loss phenotype (Figure 6D), suggesting that the *Ctr9^RNAi^* phenotypes do not depend on the genetic background of a specific Gal4 driver.

Finally, we induced *Ctr9^KO^* clones during development using *nos-Gal4* to drive expression of FLP within developing germ cells. This approach should induce homozygous *Ctr9* mutant germ cell clones with timing similar to the RNAi-induced *Ctr9* knockdown. We observed a low percentage of germaria with clones 4 d after eclosion (Figure 6E), reflecting the low level of mitotic divisions within primordial germ cells. The percentage of clonal germaria continued to increase during the first 7 d post eclosion and then plateaued. Importantly, we never observed developmentally-induced *Ctr9^KO^* germ cell clones that displayed defects in nuclear morphology (Figure 6, F and G). However, similar to the phenotype observed in *hs-FLP*-induced clones, *nos-Gal4 > UAS-FLP*-induced *Ctr9* germ line clones resulted in egg chamber degeneration during midoogenesis (Figure S4). Together, these various lines of evidence suggest that differences in the timing of the loss of *Ctr9* function does not account for differences between the *Ctr9^RNAi^* and *Ctr9^KO^* phenotypes.

## Discussion

While previous efforts have identified and characterized a number of components of the *Drosophila* Paf1 complex, a clear homolog of Ctr9 remained missing. Here, we identify *Drosophila* CG2469 as a functional homolog of human CTR9. This assessment is based on amino acid sequence similarity throughout the proteins, and is further supported by the observation that the product of a human *CTR9* cDNA transgene largely colocalizes with endogenous *Drosophila* Ctr9 within germ cells. In addition, this human cDNA transgene rescues phenotypes associated with both *Ctr9^RNAi^* knockdown and the *Ctr9^KO^* allele. Of note, *Ctr9^KO^* flies rescued by the human transgene remain sterile. By contrast, *Ctr9^KO^* flies expressing a *Drosophila* transgene inserted into the same landing site as the human transgene appear fully viable and fertile. These results suggest that many, but not all, molecular functions are conserved between *Drosophila* and human Ctr9.

The *Ctr9^RNAi^*-induced phenotypes do not match those caused by loss-of-function mutations in the gene. Reducing levels of *Ctr9* using three independent RNAi transgenes results in defects within germ cell nuclei. This phenotype can be rescued by coexpressing a presumably nontargetable human cDNA transgene. However, germline clones of a *Ctr9* null mutation appear to proceed through the early steps of cyst development without obvious defects in nuclear morphology. We have not been able to determine how knockdown by *Ctr9^RNAi^* transgenes results in a presumptive polyploidization phenotype whereas mutations in the gene do not. We have considered various mechanisms. Several experiments, including the adult-specific expression of RNAi and the developmental induction of *Ctr9^KO^* clones, suggest that the timing of *Ctr9* disruption does not account for the observed differences. Specific off-target effects also appear unlikely, given that we have used three independent RNAi transgenes and rescued the phenotypes with a nontargetable rescue construct. However, we cannot completely exclude this possibility. It may be that the induction of an RNAi pathway response in the absence of *Ctr9* causes the observed germ cell defects. Further experiments will be needed to determine the underlying cause of these phenotypes. However, the data presented here should serve as a cautionary tale about the sole use of RNAi in evaluating the genetic functions of any given gene, especially with the recent development of CRISPR/Cas9 methods for creating genetic lesions within a given locus.

We also demonstrate that deletion of *Ctr9* does not affect GSC and FSC maintenance. Previous studies, examining the loss of Bre1 in the germline, suggested a strong correlation between H3K4me3 levels and the maintenance of germline stem cells. We confirmed that loss of *Bre1* results in defects in GSC maintenance, as reported. Further work characterizing the transcriptomes and epigenomes of different Paf1 complex member mutants will provide further clarification regarding how these proteins interact and coordinate with one another within different contexts.

## Supplementary Material

Supplemental Material
