# Supplementary material for: Drosophila CG2469 Encodes a Homolog of Human CTR9 and Is Essential for Development
Source: G3 (Bethesda). 2016 Sep 27;6(12):3849–57. doi: 10.1534/g3.116.035196 (PMC5144956; doi:10.1534/g3.116.035196)
Supplement: Supplemental Material [file supp_6_12_3849__index.html]

Drosophila CG2469 Encodes a Homolog of Human CTR9 and Is Essential for Development — Supplemental Material 

# *Drosophila CG2469* Encodes a Homolog of Human CTR9 and Is Essential for Development

## Supplemental Material for Chaturvedi *et al.*, 2016

**Files in this Data Supplement:**

- Figure S1 - Human CTR9 rescues *Drosophila* Ctr9 homozygous lethality. (.pdf, 3 MB)
- Figure S2 - *nos*-Gal4 driven RNAi lines display consistent nuclear morphology phenotypes in germaria and show loss of H3K4 trimethylation in the germline. (.pdf, 3 MB)
- Figure S3 - *Drosophila* and human transgenes of *Ctr9* can rescue the nuclear defects caused by expression of *Ctr9RNAi*. (.pdf, 2 MB)
- Figure S4 - Germline clones of *Ctr9KO* degenerate at stage 8/9. (.pdf, 1 MB)
- Table S1 - Primers used to generate Ctr9KO allele. (.xlsx, 27 KB)
