# Supplementary material for: Drosophila CG2469 Encodes a Homolog of Human CTR9 and Is Essential for Development
Source: G3 (Bethesda). 2016 Sep 27;6(12):3849–57. doi: 10.1534/g3.116.035196 (PMC5144956; doi:10.1534/g3.116.035196)
Supplement: Supplemental Material [file supp_g3.116.035196_FigureS1.pdf]

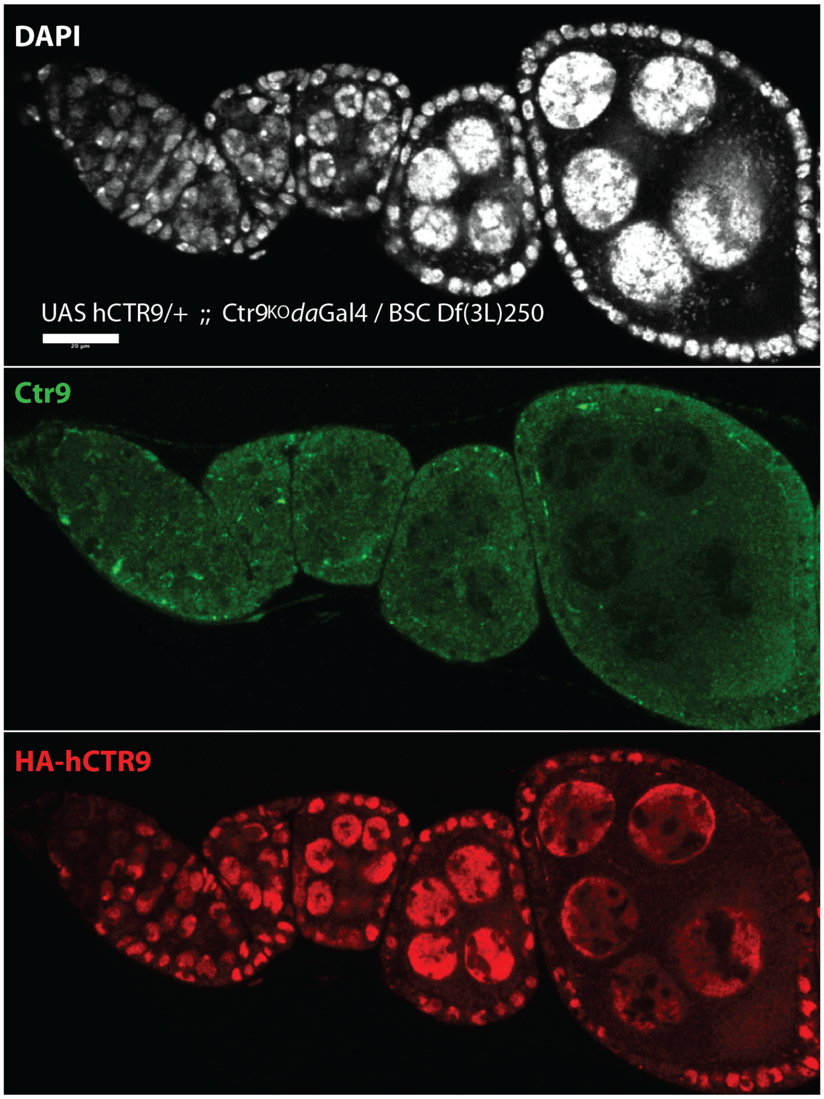

Fig. S1. Human CTR9 rescues *Drosophila* Ctr9 homozygous lethality. Ubiquitous expression of human HA tagged Ctr9 (red) driven by *daughterless*-Gal4 rescues homozygous lethality of *Ctr9*^{KO}/ *Df(3L)BSC250*. No defect in the morphology of ovaries was observed. The antibody against *Drosophila* Ctr9 shows no signal in rescue ovarioles (green). However, HA tagged human CTR9 (red) is readily detected, although not expressed at high levels in all cells.
