# Supplementary material for: Drosophila CG2469 Encodes a Homolog of Human CTR9 and Is Essential for Development
Source: G3 (Bethesda). 2016 Sep 27;6(12):3849–57. doi: 10.1534/g3.116.035196 (PMC5144956; doi:10.1534/g3.116.035196)
Supplement: Supplemental Material [file supp_g3.116.035196_FigureS2.pdf]

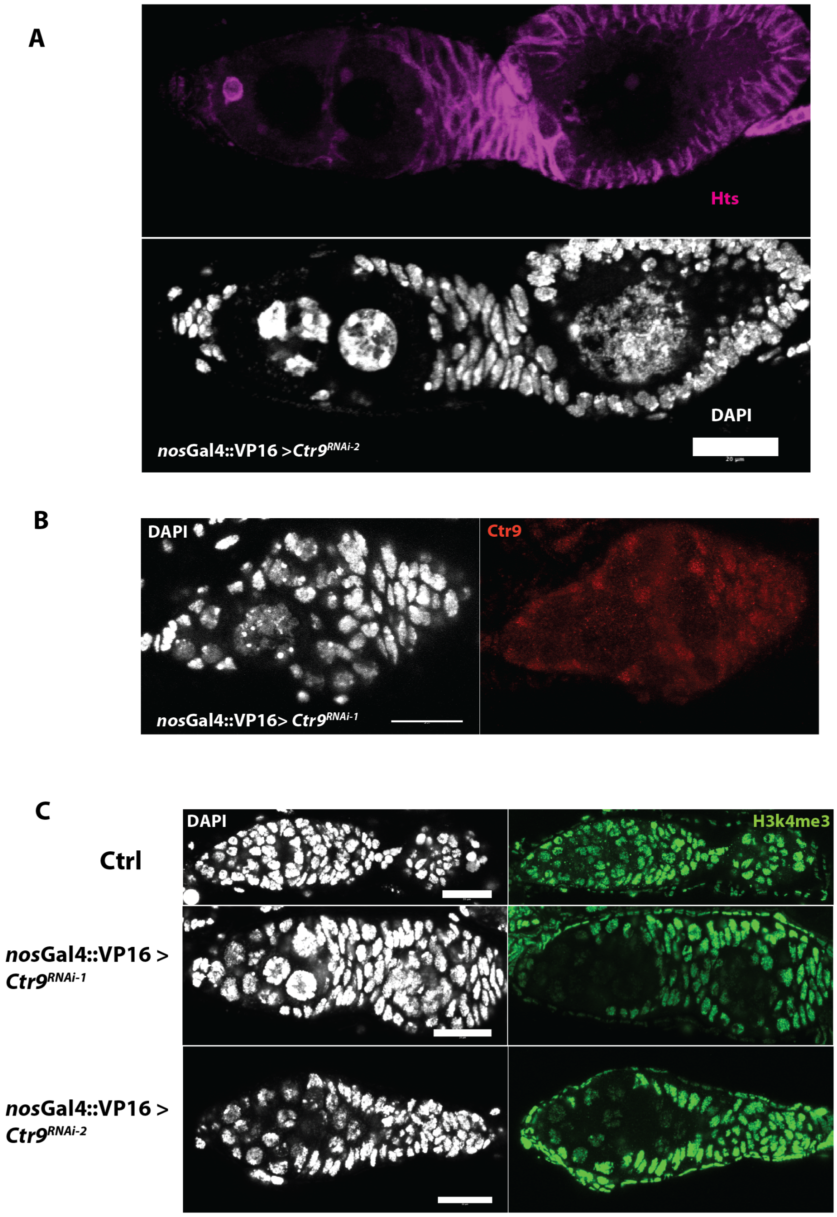

Fig. S2. *nos*-Gal4 driven RNAi lines display consistent nuclear morphology phenotypes in germaria and show loss of H3K4 trimethylation in the germline. (A) Ovarioles from *nos*-Gal4>*Ctr9*$^{RNAi\text{-}2}$ females at Day 14 stained for Hts (magenta) and DAPI (gray). Several germ cells that exhibit the premature polyploid phenotype can be observed. (B) Ovarioles from *nos*-Gal4>*Ctr9*$^{RNAi\text{-}1}$ females stained for Ctr9 (red) and DAPI (gray). A large polyploid germ cell is

seen in the germarium. (C) Control and *Ctr9*^{RNAi} samples stained for H3K4me3 (green) and DAPI (gray). Germ cells in WT germaria display H3K4 trimethylation, while *Ctr9*$^{RNAi-1}$ and *Ctr9*$^{RNAi-2}$ knockdown within the germline results in decreased H3K4me3 staining. Scale bar represents 20μm.
