# Supplementary material for: Drosophila CG2469 Encodes a Homolog of Human CTR9 and Is Essential for Development
Source: G3 (Bethesda). 2016 Sep 27;6(12):3849–57. doi: 10.1534/g3.116.035196 (PMC5144956; doi:10.1534/g3.116.035196)
Supplement: Supplemental Material [file supp_g3.116.035196_FigureS3.pdf]

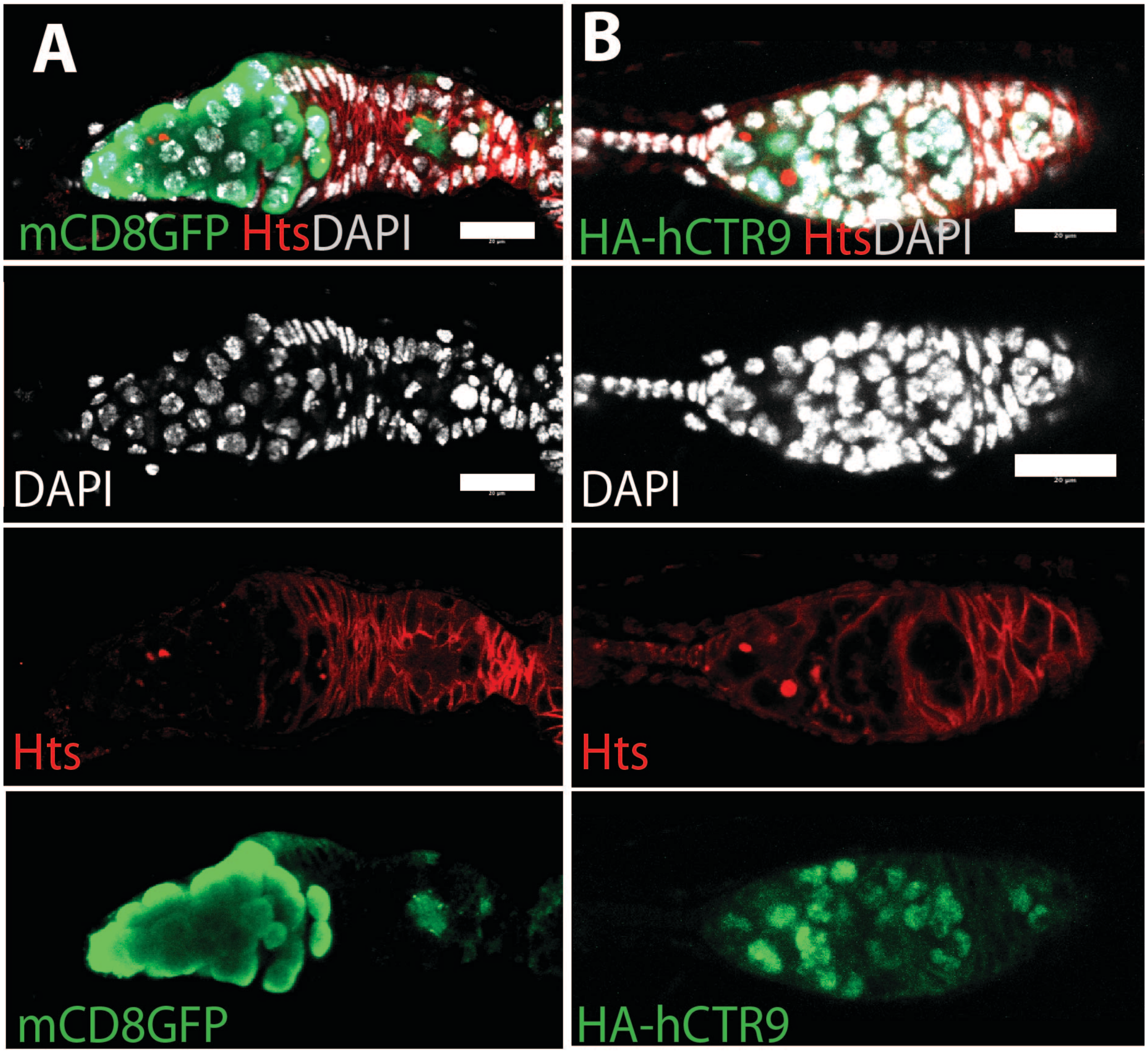

Fig. S3. ***Drosophila*** **and human transgenes of** ***Ctr9*** **can rescue the nuclear defects caused by expression of** ***Ctr9^{RNAi}*****.** (A) *nos-Gal4::VP16>Ctr9^{RNAi-1}* , *UAS-mCD8-GFP* germarium stained for GFP (green), Hts (red) and DAPI (gray). (B) Concomitant over-expression of HA tagged human Ctr9 (green) with *nos-Gal4::VP16>Ctr9^{RNAi-1}* can restore the normal nuclear morphology of germ cells (gray) with 100% penetrance. (A) Scale bars represent 20μm.
