# Supplementary material for: Drosophila CG2469 Encodes a Homolog of Human CTR9 and Is Essential for Development
Source: G3 (Bethesda). 2016 Sep 27;6(12):3849–57. doi: 10.1534/g3.116.035196 (PMC5144956; doi:10.1534/g3.116.035196)
Supplement: Supplemental Material [file supp_g3.116.035196_FigureS4.pdf]

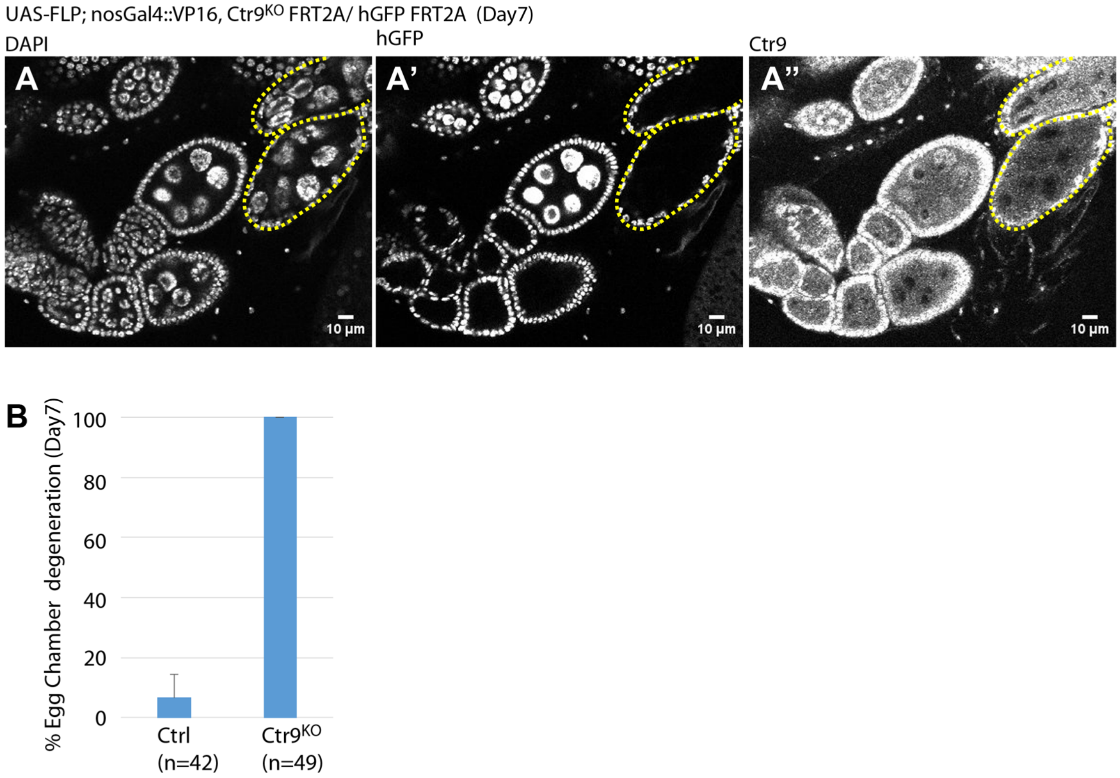

Fig. S4. Germline clones of *Ctr9^{KO}* degenerate at stage 8/9.

(A) Representative image of *Ctr9^{KO}* clonal egg chambers (Encircled by dotted line, KO clones are GFP negative), which exhibit stage 8/9 egg chamber degeneration. Ovaries were dissected at day 7 after eclosion. (B) Quantification of stage 8/9 egg chamber degeneration of *Ctr9^{KO}* clones compared with control clones at day 7 after eclosion (n=number of scored clone egg chambers). Scale bars: 10μm. *** P ≤ 0.001.
